# Occupation Associated with Altered Steroid Hormone Metabolism in Men: A Cross-Sectional Metabolome-Wide Association Study

**DOI:** 10.21203/rs.3.rs-9476065/v1

**Published:** 2026-04-22

**Authors:** Alia R. Bly, Nickilou Krigbaum, Piera Cirillo, ViLinh Tran, Mary Nellis, Young-Mi Go, Xu Liu, Caitlin C. Murphy, Dean Jones, Barbara Cohn, Xin Hu

**Affiliations:** Emory University; Child Health and Development Studies, Public Health Institute; Child Health and Development Studies, Public Health Institute; Emory University; Emory University; Emory University; Emory University; University of Chicago; Emory University; Child Health and Development Studies, Public Health Institute; Emory University

**Keywords:** Occupational Health, Crafts and Labor, Metabolomics, Steroid Hormones

## Abstract

**Objectives.:**

This exploratory study aims to characterize metabolic distinctions among men in crafts and labor occupations hypothesized to underlie the shared risk of chronic diseases observed among occupational groups historically referred to as blue collar.

**Methods.:**

Using high-resolution mass spectrometry, we characterized the serum metabolome of 220 men (n = 98 vs. 122, craftsmen and laborers vs. others) enrolled in the founding generation (1959–1967) of the Child Health and Development Studies. Metabolic changes associated with occupation were identified using Student’s t-tests and multivariable linear regression models, followed by pathway enrichment analysis.

**Results.:**

Alterations in steroid hormone metabolism were consistently found among craftsmen and laborers. The abundance of C21-steroids, such as tetrahydrocorticosterone, were increased in craftsmen and laborers, whereas C19-steroids, including androstenedione, testosterone, and 5-dihydrotestosterone, were reduced. Alteration of energy metabolism was also evidenced by reductions in acylcarnitines. In addition, several metabolic pathways such as androgen synthesis, carnitine shuttle, and bile acid synthesis showed BMI-dependent differences.

**Conclusion.:**

Alterations in steroidogenesis and energy metabolism observed in serum samples from male craftsmen and laborers offer insights into the biological pathways that may contribute to increased health risks in both these workers and their offspring. These findings provide a framework for elucidating shared metabolic responses associated with a suite of occupational conditions characteristic of craftsmen and laborers.

## 1) Introduction

Occupation has long been recognized as a major contributor to disease and premature mortality. In the year 2016, an estimated 1.9 million deaths were attributed to occupational risk factors, the majority due to non-communicable diseases.[1] Men in crafts, operative, and labor occupations, historically referred to as blue collar workers, face disproportionate risks for chronic disease and lower life expectancy.[2] There is a sizable literature on the relation of men’s work to external exposures and health hazards; however, it is typically limited to one occupation or exposure class at a time[3–6], leaving the shared elevated disease risks across diverse industries unexplained. While workers are exposed to numerous occupational stressors, prior literature indicates that only a subset of resulting biological responses ultimately dictate disease susceptibility.[7] These molecular phenotypes can now be interrogated as predictive characteristics of future disease risk.[8, 9]

Metabolomics provides a powerful approach for characterizing molecular signatures of exposure response because metabolites—endogenous small molecules—represent the functional output of biological machinery.[10] Advances in high-resolution mass spectrometry (HRMS) now enable comprehensive profiling of metabolites across more than 100 biological pathways.[11] Such assembly of omics scale metabolic data has been successfully used to identify early molecular predictors of disease within the same individuals and, in certain contexts, across generations.[12, 13] Fetal programming of metabolism, whereby the maternal and/or paternal environment has lasting effects on offspring health, has previously been established through measurement of the maternal periconceptual exposome.[14, 15]

In the present study, we analyzed serum samples from men who fathered pregnancies in the Child Health and Development Studies (CHDS) pregnancy cohort between 1959 and 1967. We previously found that the offspring of the CHDS fathers in crafts, operative, service, or labor occupations had a 2-fold increase in colorectal cancer risk in adulthood.[16] Here, we conducted a metabolome-wide association study (MWAS) among a subset of CHDS fathers with available biospecimens who had been selected for a prior study to identify metabolic signatures distinguishing craftsmen and laborers from professional, technical managerial, clerical, and sales workers. These findings aim to identify shared molecular pathways relevant to male craftsmen and laborers, providing opportunities for individualized prevention strategies and generation of hypotheses for future investigation of intergenerational health risk associated with paternal occupation.

## 2) Methods

### 2.1) Data and Blood Collection

The Child Health and Development Studies Cohort (CHDS) enrolled families residing in the Oakland, California area, all of whom were members of the Kaiser Foundation Health Plan, that included couples with pregnant women receiving obstetric care from 1959 to 1967.[17] Interviews were conducted with the pregnant women at enrollment to collect data regarding their husbands’ height, weight, highest level of completed education, occupation, income, smoking and alcohol consumption habits, and additional lifestyle factors. Paternal occupation was subsequently categorized per the 1977 U.S. Department of Labor’s Dictionary of Occupational Titles.[16, 18] Examples of paternal occupations classified within each occupational group are provided in Table S1. The distribution of major categories in the CHDS is similar to national estimates from the 1960 Census and the 1965 Current Population Survey.[19] A prior publication based on CHDS found that the offspring of men who were craftsmen and laborers had an increased risk of colorectal cancer.[16] Therefore, we used the same categories. We combined crafts, operative, service, or labor occupations and refer to them as craftsmen and laborers. This group was compared to the combined category of professional, technical, managerial, clerical, or sales workers.

Blood samples from the husbands collected during the pregnancies of enrolled families and stored at −20°C at the National Institutes of Health.[17] The current study sample is a convenience subsample derived from a previous study (The Chemical Safety Study) designed to examine inter and trans-generational health impacts of environmental exposure.[20, 21] For this study, we selected all founding generation males from the Chemical Safety Study with available metabolomics data, occupational and sociodemographic information, and health data on two progeny generations (n = 220). A flowchart including eligibility criteria for the current study sample is included in Figure S1.

### 2.2) High-Resolution Mass Spectrometry (HRMS) Based Metabolomics

HRMS analyses were conducted at the Emory Clinical Biomarker Laboratory as previously described. [22–24] Serum samples were treated with ice-cold acetonitrile containing an internal standard solution including fourteen stable isotopic chemicals. After chilled equilibrium and centrifugation, untargeted analysis was conducted using a Q-Exactive HF mass spectrometer (Thermo Fisher Scientific) with an electrospray ionization (ESI) source. Samples were analyzed in triplicate, and pooled reference plasma standards were analyzed in parallel to study samples within each batch.

HRMS data were collected using dual column chromatography: Hydrophilic Interaction Chromatography (HILIC) paired with positive ionization and C18 chromatography paired with negative ionization, generating HILIC-positive and C18-negative data, respectively. A scan range of 85 − 1,275 *m/z* (mass-to-charge ratio) was used. Following LC-HRMS analysis, raw instrument files were extracted and converted to mzXML files using the apLCMS package and processed using xMSanalyzer.[25, 26] Each metabolic feature was defined by a unique combination of *m/z* and retention time (rt, seconds). Metabolite intensity values from the triplicates were median-summarized and batch-effect corrected using ComBat. [27] Only metabolic features with a Pearson correlation coefficient of 0.7 or greater among technical triplicates were included in downstream analyses.

Metabolite annotation and confirmation was conducted using xMSannotator referencing the Human Metabolome Database (https://hmdb.ca/) with a tolerance of five parts per million (ppm).[28] The identities of select metabolites were confirmed by an accurate match of *m/z* and co-elution with authentic chemical standards when available through an in-house chemical library in alignment with the Level 1 criteria conceptualized by Metabolomics Standards Initiative.[29]

### 2.3) Statistical and Pathway Analysis

Metabolic features detected via the HILIC-positive and C18-negative modes were analyzed separately. Data pre-processing and preliminary statistical analyses were conducted in Rodin, an integrative web interface (https://rodin-meta.com/) and Python library that streamlines the metabolomics data workflow. [30] Data were filtered to include only features detected in more than 50% of all samples. Remaining HILIC-positive (n = 6,654) and C18-negative (n = 6,136) features were quantile normalized, log_2_-transformed, and standardized by unit variance.

Features were compared between groups using two approaches: a Student’s t-test and a multivariable linear regression model designed in R Statistical Software (v4.4.1, RStudio, Inc.). The multivariable model was adjusted for *a priori* confounders including age, race (black participants vs. non-black participants), and highest level of education completed (high school or less vs. greater than high school).

Body mass index (BMI) was not included in the multivariable model due to a high percentage of data missingness (30%). We instead conducted BMI-stratified Student’s t-tests to control for the confounding effect of BMI on the metabolome. Participants with BMI data (n = 158) were stratified by BMI < 25 kg/m^2^ (n = 105) and BMI ≥ 25 kg/m^2^ (n = 53).

To further explore functional implications of metabolic profiles among craftsmen and laborers, we conducted pathway enrichment analysis using the *mummichog* algorithm (v2.7.0).[31] Following pathway enrichment analysis, the transformed peak intensities of select metabolites with probable adducts (M + H, M-H, M + Na, or M+H_2_O) from significantly enriched pathways were visualized in RStudio. Additional information regarding annotated metabolites is available in Table S2 of Supporting Information. All models utilized an α-value of 0.05 to establish statistical significance kand further underwent pathway enrichment analyses using *mummichog*.[31] This approach protects against type 2 statistical error by including all features at a raw *p-value* < 0.05 while protecting against type 1 statistical error by permutation testing used in pathway enrichment analysis.

## 3) Results

### 3.1) Study Characteristics

Table 1 summarizes characteristics of the 220 male CHDS participants. Craftsmen and laborers comprised 44.5% of the sample, specifically 29.5% crafts or operative and 15.0% service work or labor, while other occupations (55.5%) included 46.4% professional, technical, or managerial, 8.6% clerical or sales, and 0.45% unemployed (Table S1). Non-white participants predominated among the craftsmen and laborers as compared to those in other occupations (p < 0.001). Furthermore, there were significant differences by education level (p < 0.001). Approximately 75% of the crafts and labor group had a high school education level or less as compared to other occupations where this proportion was reversed. Notably, no one in the crafts and labor group reported completion of college versus 65% of participants in the other occupational groups. Despite missing data, BMI data was available in similar proportion for both occupational groups.

### 3.2) Unadjusted Model of Occupational Status and Metabolic Profile

**Figure 1:**
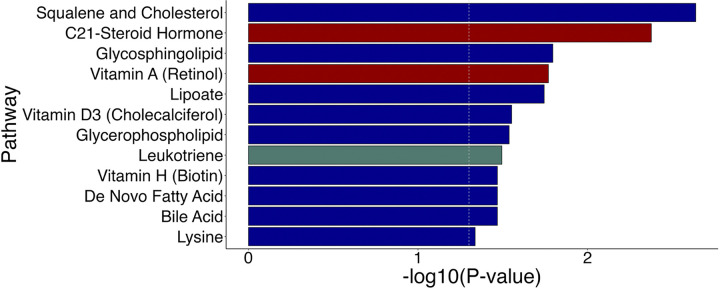
Pathway enrichment analysis identified metabolic signatures different between craftsmen and laborers and participants of other occupations. Color coding indicates if pathways were enriched among features detected in HILIC-positive mode only (blue), C18-negative mode only (green), or both ionization modes (red). Analysis was conducted using *mummichog* with the x-axis indicating the significance of the pathway.

We found 489 HILIC-positive and 539 C18-negative metabolic features significantly altered among craftsmen and laborers relative to men in other occupations (Student’s t-test, p < 0.05). These features represented metabolic pathways related to sterol and lipid homeostasis, such as squalene and cholesterol biosynthesis, C21-steroid hormone biosynthesis and metabolism, glycosphingolipid metabolism, vitamins A (retinol) and D3 (cholecalciferol) metabolism, bile acid biosynthesis, and leukotriene metabolism (Fig. 1).

**Figure 2:**
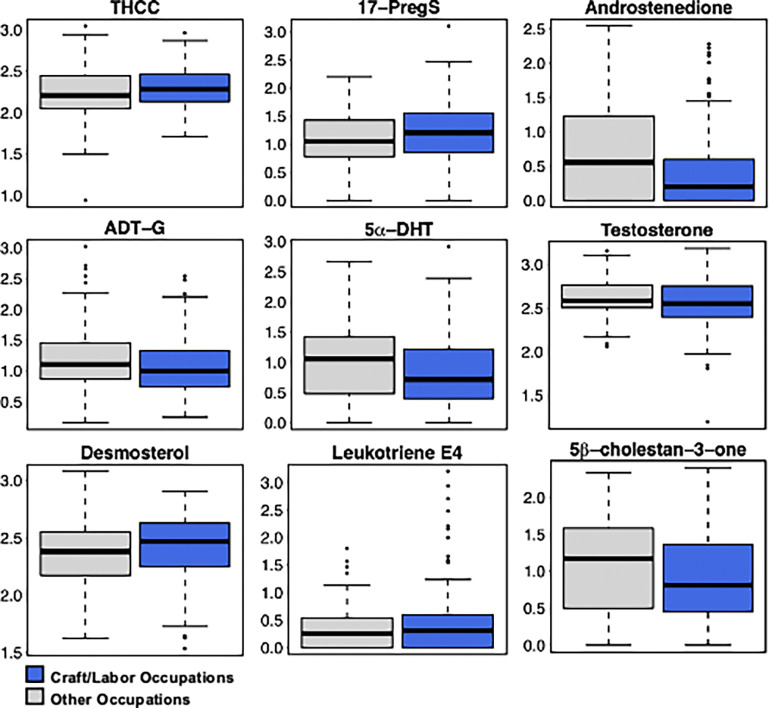
Metabolites different between occupational groups selected from enriched metabolic pathways. Student’s t-test *p-values* of the selected metabolites were 0.03 (tetrahydrocorticosterone [THCC], *m*/*z* 349.2362, rt 281 s), 0.04 (17a-hydroxypregnalone-sulfate [17-PregS], *m/z* 410.1797, rt 65.8 s), 0.003 (androstenedione, *m/z*309.1799, rt 67.4 s), 0.03 (androsterone glucuronide [ADT-G], *m/z*465.2488, rt 40.3 s), 0.006 (5a-dihydrotestosterone [DHT], *m/z* 289.2173, rt 33.3 s), 0.3 (testosterone, *m/z* 307.2254, rt 26.3 s), 0.04 (desmosterol, *m/z* 385.3455, rt 27.8 s), 0.01 (leukotriene E4, *m/z* 466.1923, rt 66.8 s), and 0.01 (5b-cholestan-3-one, *m/z*409.3458, rt 236.3 s).

Within those pathways, desmosterol (the cholesterol precursor), the pro-inflammatory leukotriene E4, the C21 steroid hormone tetrahydrocorticosterone (THCC), and 17α-hydroxypregnalone-sulfate (17-PregS) increased, while androstenedione, androsterone glucuronide (ADT-G), and 5α-dihydrotestosterone (DHT) were decreased in craftsmen and laborers (Fig. 2). While testosterone was not significantly different between the occupational groups, the relative abundance was decreased among craftsmen and laborers, consistent with the broader trend of reductions in androgens. These changes indicated a broad disruption in cholesterol derived steroid hormone synthesis, in particular the C21 glucocorticoids and C19 androgens.

### 3.2) Multivariable Model of Occupational Status and Metabolic Profile

We further investigated the metabolome associations with craft and labor occupations using a multivariable linear regression model adjusted for age, race/ethnicity, and education as potential confounders. The model identified 393 and 447 significantly different (p < 0.05) metabolites in HILIC-positive and C18-negative data, respectively. Enriched pathways were, in general, consistent with the unadjusted model, including glycerophospholipid and C21-steroid hormone biosynthesis and metabolism (Figure S2).

After adjusting for covariates, the carnitine shuttle, a central component of energy metabolism responsible for transporting long-chain fatty acids across the mitochondrial membrane, was among the top changed pathways. Five acyl-carnitines (i.e., intermediates responsible for transporting fatty acids into mitochondria for metabolism[32]) were negatively associated with crafts or operative and service work or labor occupations (Figure S3). In addition, the C19-hormone testosterone was negatively associated with these occupations (Figure S3). C21 steroids including aldosterone and progesterone showed an increasing yet non-significant trend (Figure S4).

### 3.3) BMI-Stratified Analysis

To remove the potential confounding effect of BMI (kg/m^2^) in our study, we stratified participants into lean (BMI < 25) and high (BMI ≥ 25) BMI groups and examined occupational effects within each group. Among the lean men, we detected 397 and 454 significantly different metabolic features between occupational groups in the HILIC-positive and C18-negative data, respectively. Pathway enrichment analysis demonstrated significant alteration of pathways consistent with the unstratified analysis including steroid hormone and vitamin A metabolism (Figure S5A).

Compared to the lean group, fewer different metabolites were found in the high-BMI group (248 and 216 in the HILIC-positive and C18-negative data, respectively), yet the number of enriched pathways was greater in this stratum. C21-steroid biosynthesis and metabolism remained significantly different among craftsmen and laborers. Also concordant with the unstratified analysis was the alteration of pathways synthesizing bile acid, including squalene and cholesterol. Unique to the high-BMI participants included enrichment of pathways related to carbohydrate metabolism, such as pentose phosphate and fructose and mannose metabolism (Figure S5B).

**Figure 3:**
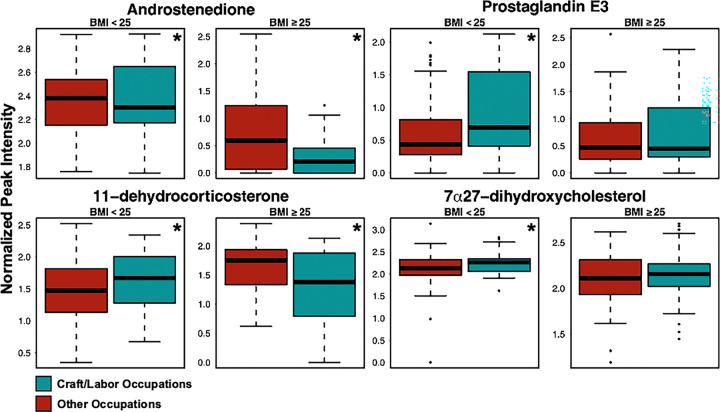
Distribution of metabolite levels for four significant features annotated as androstenedione (*m*/*z* 309.1799, rt 67 s), 11-dehydrocorticosterone (*m/z*345.208, rt 27.3 s), 7a, 27-dihydroxycholesterol (*m/z* 419.352, rt 26.3 s), and PGE3 (*m/z* 349.2017, rt 92.3 s). Values are presented as transformed peak intensity in boxplots. An asterisk is used to denote comparisons that were significantly different (p < 0.05) between the occupational groups in each BMI stratum.

Within the enriched pathways, we found metabolites that were concordantly and discordantly abundant across the BMI strata. For example, craftsmen and laborers had reduced levels of androstenedione across both BMI strata (p < 0.05). Levels of 7 *α*, 27-dihydroxycholesterol (a bile acid precursor) trended consistently higher in craftsmen and laborer, although only significant in the lean group. Similarly, Prostaglandin E3 (PGE3) was elevated only within the lean group (Fig. 3).

**Figure 4:**
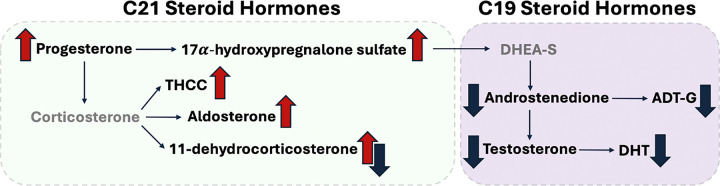
A summary of metabolites from the steroid hormone biosynthesis pathway with blue arrows indicating decreased abundance in craftsmen and laborers, red arrows indicating increased abundance, and paired blue and red arrows indicating a discordant trend based on BMI stratification. Metabolites labeled in grey were not identified our data.

In contrast, 11-dehydrocorticosterone was significantly changed across both BMI strata, but the directions were opposite: higher in craftsmen and laborers in the lean group and lower in craftsmen and laborers in the high-BMI group. These results demonstrated shared alteration of pathways and constituent metabolites central to endocrine function while revealing BMI-specific effects in craftsmen and laborers (Fig. 4).

A full list of differentially abundant metabolites across BMI strata in pathways such as C21-steroid hormone biosynthesis, androgen and estrogen metabolism, squalene and cholesterol metabolism, and glycerophospholipid metabolism is available in Table S3.

## 4) Discussion

Extensive literature links occupational exposures to adverse health outcomes, particularly among workers historically classified as blue collar, yet molecular characterization of these associations remains limited. Identifying endogenous biomarkers or reprogrammed pathways that reflect individual biological responses to occupational environments is key to understanding risk mechanisms and improving prevention strategies. People in crafts, operative, and labor occupations are typically exposed to an array of chemical and physical hazards, and single-exposure analyses cannot fully account for the shared elevation in health risks or inform feasible risk mitigation. Our prior work demonstrated that diverse environmental exposures converge on common metabolic pathways[7, 33], providing a framework to use metabolomics for defining molecular phenotypes that predict long-term health trajectories. Building on this foundation, the present metabolome-wide association study (MWAS) identifies metabolic profiles associated with those high-risk occupations. These findings may have implications for multiple chronic health outcomes including reproduction, cancer, cardiovascular disease, and neurological disorders.

We found strikingly consistent alterations in steroid biosynthesis and lipid metabolism among craftsmen and laborers. The C21 steroids hormone biosynthesis pathway, which includes glucocorticoid and mineralocorticoid molecules such as cortisol and aldosterone that regulate stress response, immune function, and metabolism[34], was the most consistently enriched pathway across all statistical models, regardless of adjustment for confounders. Notably, tetrahydrocorticosterone, a reduced product of corticosterone (a glucocorticoid hormone produced by the adrenal cortex), was consistently elevated in craftsmen and laborers. Its elevation may be clinically interpreted as an indication of excessive adrenal activity, often associated with chronic stress or of the hypothalamic–pituitary–adrenal (HPA) axis dysregulation.[34] This pattern was further supported by a marginal increase in aldosterone (synthesized by corticosterone), suggesting broader alterations in adrenal steroidogenesis associated with blue-collar occupations of crafts, operative, service work and labor.

An intriguing BMI-dependent contrast was observed for 11-dehydrocorticosterone, another inactive corticosterone metabolite. Levels were higher in lean craftsmen and laborers compared to their counterparts of other occupations, yet lower in high-BMI craftsmen and laborers. This differential response may reflect variation in 11β-hydroxysteroid dehydrogenase (11β-HSD) activities which are responsible for the interconversion between the active glucocorticoid (e.g., cortisol and corticosterone) and the inactive forms (e.g., cortisone and 11-dehydrocorticosterone). The type 1 (11β-HSD1) enzyme specifically reactivates 11-dehydrocorticosterone back to corticosterone and is highly expressed in all adipose tissue depots[35], which may explain lowered level of 11-dehydrocorticosterone in high-BMI craftsmen and laborers. This example illustrates the utility of metabolomics in capturing individualized biological responses to occupational environments.

We also found widespread disruption of the C19 androgens, which are directly synthesized from the C21 steroids. 17α-hydroxypregnenolone sulfate (17OH-PregS), synthesized by the adrenal glands, serves as a stable, circulating source of precursors for androgen production. It can be converted to dehydroepiandrosterone sulfate (DHEA-S) and transported to peripheral tissues for androgen synthesis. Despite elevated levels of 17OH-PregS, we found consistently reduced levels of androgens in craftsmen and laborers, including testosterone (T), androstenedione (a direct precursor of T), DHT (a potent derivative of T), as well as ADT-G (a major circulating and urinary metabolite of both T and DHT).[36] These changes indicate a broad decrease in androgen steroidogenesis that is consistent with the pattern of adrenal hyperactivity and endocrine dysregulation seen with C21 steroids.

Altered steroidogenesis was further substantiated by disruptions in upstream cholesterol and lipid metabolism pathways, such as squalene and cholesterol biosynthesis, and other cholesterol-derived biosynthetic pathways such as bile acid biosynthesis and vitamin D metabolism. The upregulation of desmosterol, a key intermediate in cholesterol biosynthesis, suggests dysregulation not only of downstream cholesterol derivatives but also of cholesterol homeostasis itself, an imbalance previously linked to adverse cardiovascular outcomes such as atherosclerosis and myocardial infarction.[37] In parallel, consistent enrichment of glycerophospholipid and vitamin A metabolism indicated an even broader perturbation of lipid metabolic networks, highlighting the systemic nature of metabolic alterations linked to crafts or operative and service work or labor occupations.

The carnitine shuttle, an integral component of mitochondrial energy metabolism, facilitates the transport of long-chain fatty acids (LCFAs) across the mitochondrial membrane for *β* -oxidation.[32] Acylcarnitines, formed by conjugation of fatty acids with carnitine, were consistently decreased among the craftsmen, including tetradecanoyl carnitine, stearoylcarnitine, palmitoylcarnitine, octadecanoyl carnitine, and heptadecanoyl carnitine. These decreases suggest impaired mitochondrial fatty acid utilization, which can lead to insufficient energy production and secondary effects such as oxidative stress and disrupted calcium homeostasis.[38] Alterations in acylcarnitine abundance and the carnitine shuttle pathway have previously been linked to occupational exposures, like benzene, via an HRMS-based MWAS, while a growing body of literature also suggests a role of environmental exposures, like pesticides and endocrine-disrupting chemicals (EDCs), in dysregulated mitochondrial energetics.[6, 39]

Given the correlative and cross-sectional design based on a convenience sample, our findings cannot establish causality, assess longitudinal effects, or be generalized to the broader population. Differences observed across occupational groups may reflect a range of holistic environmental and behavioral differences (e.g., working hours, physical activity, dietary intake, occupational exposures). [1] Thus, occupation may serve as a proxy for the broader environment of the working class. Nonetheless, our results provide evidence that craftsmen and laborers as a whole are at increased risk for adverse health outcomes associated with steroid dysregulations. Because the first CHDS generation was recruited from 1959 and 1967, the occupational exposure landscape at that time may differ from that of modern workplaces. However, many occupational hazards during that period remain in use or persist in the environment, and craftsmen and laborers today continue to face heightened health risks.[40] The metabolic profiles observed in this generation therefore provide valuable mechanistic insights relevant to current challenges in occupational health and safety. Importantly, prior work in the CHDS cohort reported a two-fold increase in colorectal cancer risk among offspring of fathers in crafts and labor occupations, suggesting potential intergenerational implications of occupation-related endocrine dysregulation. The longitudinal and multi-generational design of the CHDS offers a unique opportunity to investigate these links more directly in future studies.

## 5) Conclusions

In summary, this HRMS-based MWAS revealed distinct metabolic profiles that differentiate craftsmen and laborers from other occupational groups at both the pathway and metabolite levels. Crafts or operative and service work or labor occupations was consistently associated with alterations in adrenal steroid biosynthesis, mitochondrial fatty acid metabolism, and cholesterol and lipid pathways. These metabolic signatures represent individualized but shared biological responses to complex exposures, with important implications for reproductive and metabolic health among craftsmen and laborers.

## Supplementary Material

Supplementary Files

This is a list of supplementary files associated with this preprint. Click to download.

- BlyOccupationMWASSIApril1.docx

## Figures and Tables

**Table 1:** Sociodemographic characteristics of participants.

Age (years)	Other occupations (N = 122)	Craftsmen and laborers (N = 98)	Overall (N = 220)	*P-value* *
			
Median (Min, Max)	28.5 (19.0, 48.0)	29.0 (19.0, 54.0)	29.0 (19.0, 54.0)	0.95
**Race/Ethnicity, N(%)**				
White	100 (82.0)	48 (49.0)	148 (67.3)	*< 0.001*
Black	9(7.4)	36(36.7)	45(20.5)	
Other	13 (10.7)	14(14.3)	27 (12.3)	
**Highest Level of Education, N(%)**				
High School or Less	17 (13.9)	73 (74.5)	90 (40.1)	*< 0.001*
Greater Than High School	105 (86.1)	25 (25.5)	130 (59.1)	
**BMI (kg/m^2^)**				
Median (Min, Max)	23.6 (19.0, 29.3)	23.6 (19.6, 34.4)	23.6 (19.0, 34.4)	0.26
Missing, N(%)	34 (27.9)	32 (32.7)	66 (30.0)	
**Overweight or obese (%)**				
BMI ≥ 25	22.1	24.5	23.2	0.80

* Student’s t-test, Fisher’s exact test, or two-proportions z-test where appropriate.

## Data Availability

De-identified (anonymized) data are available upon request from the corresponding author. Requests will be reviewed by Barbara A. Cohn (Director of the Child Health and Development Studies), the research staff, and the Institutional Review Board of the Public Health Institute. Approval of requests for de-identified data required execution of a data use agreement.

## What is already known on this topic

Craftsmen and laborers experience higher risk for chronic disease than men in other occupations, and this risk may even transmit to the next generation. Many individual occupational factors, including chemical and physical hazards, socioeconomic disadvantage, physical demands, and psychosocial stress, have been implicated, yet the shared biological mechanisms through which diverse occupationally related factors contribute to increased health risk remain poorly understood.

## What this study adds:

This study provides a comprehensive metabolome-wide characterization of molecular differences among craftsmen and laborers compared with men in other professions. By capturing integrated biological responses to complex occupational environments, the study identifies candidate biological pathways that may underlie the elevated chronic disease risk observed among working-class men.

## How this study might affect research, practice, or policy:

Improved understanding of biological susceptibility in occupational health research can inform targeted prevention strategies and more precise assessment of occupation-related disease risk among working populations. Discovery of personal biomarkers to define disease risk can provide opportunities for targeted early intervention in the populations of working men.
